# Spatial Structures of the Environment and of Dispersal Impact Species Distribution in Competitive Metacommunities

**DOI:** 10.1371/journal.pone.0068927

**Published:** 2013-07-18

**Authors:** Dexiecuo Ai, Dominique Gravel, Chengjin Chu, Gang Wang

**Affiliations:** 1 State Key Laboratory of Grassland and Agro-Ecosystems, School of Life Sciences, Lanzhou University, Lanzhou, China; 2 Département de Biologie, Chimie et Géographie, Université du Québec à Rimouski, Rimouski, Canada; University of Florida, United States of America

## Abstract

The correspondence between species distribution and the environment depends on species’ ability to track favorable environmental conditions (via dispersal) and to maintain competitive hierarchy against the constant influx of migrants (mass effect) and demographic stochasticity (ecological drift). Here we report a simulation study of the influence of landscape structure on species distribution. We consider lottery competition for space in a spatially heterogeneous environment, where the landscape is represented as a network of localities connected by dispersal. We quantified the contribution of neutrality and species sorting to their spatial distribution. We found that neutrality increases and the strength of species-sorting decreases with the centrality of a community in the landscape when the average dispersal among communities is low, whereas the opposite was found at elevated dispersal. We also found that the strength of species-sorting increases with environmental heterogeneity. Our results illustrate that spatial structure of the environment and of dispersal must be taken into account for understanding species distribution. We stress the importance of spatial geographic structure on the relative importance of niche vs. neutral processes in controlling community dynamics.

## Introduction

The relationship between species distribution and the environment is a central topic in ecology [1]–[5] and is important to various fields in ecology such as biogeography, evolution and, more recently, in conservation biology and climate change research [6]–[12]. The central aspect of this relationship for modelers is obviously its strength – that is, how frequently species inhabit their most favorable conditions, or the other way around, how frequently a given location is occupied by the most competitive species [4]. It is however unrealistic that species are optimally distributed in their most suitable conditions because dispersal often constrains the potential of species from accessing some habitable areas [13] and biotic interactions, including human impacts, may further prevent establishment in some areas [4], [14]. Emergent ecological drift could also blur the relationship between distribution and the environment despite strong niche differentiation [15].

Metacommunity theory has advanced understanding of how spatial dynamics and local interactions shape community structure and biodiversity [16]–[18]. It tells us that the correspondence between species distribution and the environment depends on species’ ability to track favorable environments (via dispersal), maintain competitive hierarchy against the constant influx of migrants (mass effect) and demographic stochasticity (ecological drift) [15], [19], [20]. We understand from theory there are two important aspects of the landscape spatial structure susceptible to impact the strength of species-sorting: the connectivity matrix (i.e. the spatial arrangement among localities and dispersal rate among them) and the environmental heterogeneity (i.e. variance and range of environmental conditions and their spatial autocorrelation). However, we only have a limited understanding of how these factors interact to affect species distribution.

A metacommunity was simply defined as a set of local communities linked by dispersal [16], [21], [22]. Dispersal was considered homogeneous throughout the landscape in most original metacommunity models [20], [23]. It is however a much more natural and convenient way to represent landscapes as a network of localities connected by dispersal [24]. An advantage of the network framework is the sophisticated set of quantitative tools available for characterizing network structure [25]–[27]. For instance, metrics such as network centrality are used to measure the contribution of node position to the importance, influence, prominence of an actor in a network. In this context, the object of interest is no longer the effect of average dispersal, but rather its variance. The network approach has been influential for the development of landscape-scale conservation plans [24], [25], [28], [29] and provides a more realistic representation of spatial dynamics [30], [31]. The metacommunity of early neutral models is often represented as a large species pool [32] and held constant during local community dynamics [33]–[35]. Recent studies on neutral dynamics in spatially explicit landscapes however shown that the spatial arrangement of localities has considerable impacts on the structure at both the local scale [36], [37] and the regional scale [38], [39]. For example, it was found that local species richness increases with the centrality of the community in the spatial network, while global diversity increases with higher connectivity and dispersal for metacommunity consisted of fewer local communities.

Niche differentiation over a spatially heterogeneous environment is often considered as the main explanation for species coexistence [40]–[43]. Niche theory predicts that species richness will increase with environmental heterogeneity. The number of species that could be packed along an environmental gradient however depends on the overlap of their niches. May and McLean [44] defined the limiting similarity as the maximal overlap of species’ utilization of resources enabling their coexistence. It was later found that metacommunity dynamics could alter significantly the limiting similarity among species [23], [45]. Dispersal limitations prevent a species to fight for all its favorable locations, thereby allowing the coexistence with a competitively inferior, but similar species. Alternatively, a competitively superior species could be displaced from a location by the constant influx (a mass effect) of propagules of an inferior species. Even if both dispersal and environmental heterogeneity has been extensively studied, their effect (and interaction) on the strength of species sorting has seldom been studied.

Ecological drift is an emergent phenomenon that could also impact significantly species distribution. It is defined as population changes arising from stochastic population dynamics [32] and can be measured as the variance in community composition (e.g. with metrics of beta-diversity) between replicated time series [45]. Ecological drift is an emergent property of community dynamics affecting species distribution and is the driver underlying species abundance distributions in the neutral theory [32], [46], [47]. In non-neutral communities, ecological drift is acting along with deterministic processes to determine the species distribution [45], [48], [49]. Ecological drift can impose a limit to similarity according to Tilman [50].

Our first objective in this study is to investigate how the landscape structure influences the relative importance of species-sorting and ecological drift. Our second objective is to examine how environmental heterogeneity can affect the relative importance of these two processes. The metacommunity is represented as a set of local communities linked by dispersal in a realistic spatial network. Local dynamics follow the lottery competition for space in a heterogeneous environment proposed by Gravel et al. [15]. We use network centrality measures to quantify the position of a node in a metacommunity [37]. We consider the impact of the variance and range of environmental conditions, and the spatial structure of the heterogeneity (variance within versus between localities).

## Model Description

We consider an individual-based, spatially explicit stochastic model of metacommunity dynamics. The metacommunity consist of *n* local communities connected by dispersal. Each local community is represented as a square lattice of size *L×L* with a torus shape, where each site is occupied by a single individual. The dynamics follow the zero-sum rule of the lottery model [32]. At each time step, an individual dies with probability *d* and empty sites are filled by recruits either drawn from the local community or the surrounding ones.

### Recruitment Dynamics

Recruitment of an individual at a site follows the lottery model used by Gravel et al. [15]. The model is however expanded to account for dispersal within and between local communities. Hence, the recruitment probability *R_i,x,t_* of species *i* in local community *t* at the site *x* is:
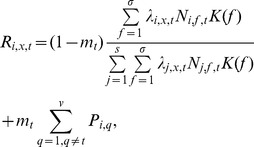
(1)where the parameter *m_t_* is the probability that a recruit is an immigrant coming from surrounding local communities connected to locality *t*. *N_j,f,t_* is the total number of individuals of species *j* at distance *f* from the site *x*. *K(f)* is the local dispersal kernel. According to Snyder and Chesson [51], short-range dispersal can facilitate coexistence when environmental variation is permanent. Hence we use the four nearest-neighbor dispersal. *P_i,q_* is the relative abundance of species *i* within the local community *q* connected to *t*. The probability of an immigrant coming from a surrounding community, *m_t_*_,_ is defined as [38]:



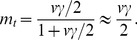
(2)Where *γ* is the weight of dispersal between local communities [52] and *v* is the number of local communities connected with *t*.

We consider a Gaussian-shaped function to describe the relationship between the environment and the survival probability *λ_i,x,t_* of offspring from species *i* at the site *x* in local community *t*:
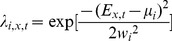
(3)where *E_x,t_* is the local environmental condition, *µ_i_* is the niche optima of species *i*, and *w_i_* is the niche breadth of species *i* (we assume for simplicity identical niche breadth for all species).

The environment is heterogeneous within and between local communities. We consider for simplicity a linear gradient of environmental conditions within each local community. The environmental condition *E_x,t_* is thus a linear function of the position on the lattice:
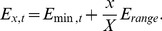
(4)


We equally divide the lattice into *X* subsections and each subsection has two dimensions. We label all subsections from 0 to *X*–1. The value of *X* thus determines the grain size of the environment. *E_x,t_* is the *E* value of section *x*, *E_range_* is the range of *E*, and *E_min, t_* is the minimal value of *E* in local community *t*. We use *E_min, t_* and *E_range_* to control the environmental heterogeneity of local community *t*. The variance of environmental conditions between local communities (regional variance) is determined by *E_range_* and *E_min,t_*. We number the local communities from 1 to *n* and *E_min,t_* is defined as *t*/*n*, for example, *E_min,0_* = 0/n, *E_min,1_* = 1/n. The variance of the environment within given local community is determined by *E_range_* and increases with *E_range_*.

The distribution of niche optima is evenly spaced across the range of available environments [43]. We number the species from 1 to *M*, with niche optima of species *i* being
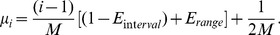
(5)where *E_interval_* is the interval of minimal environmental values between adjacent local communities, and is *E_min, t_*−*E_min, t−1_*, that is *1*/*n*. *E_range_* determines the similarity among niche optimums.

### Network Construction and Centrality Measures

The metacommunity is represented by a graph where each of the *n* local communities is considered a node. We use the random geometric graph algorithm [53] to generate the network structure. Unlike simple shapes (such as circle and star) and random graphs, the random geometric graph has a heterogeneous degree distribution and represents landscapes more realistically. A random geometric graph is constructed by dropping *n* points randomly into a unit square and by connecting any two points within a threshold distance (Euclidean) *r* from each other. We ensure that the network is fully connected (a single graph). We assume equal weights for all edges.

Typical network studies address issues of connectivity and centrality. Connectance (the fraction of realized links between nodes relative to all potential links) is a network-level property quantifying the average connectivity of the network. Centrality is a node-level property, quantifying its position relative to other nodes in the network. We consider a variety of network centrality measures to measure different aspects of network topology. Betweenness centrality is the number of shortest paths that the focal node lies on [52] in other words the number of ‘times’ that any node needs a given node to reach any other node by the shortest path. This index is a useful measure of the node’s importance to the network [54]. Closeness centrality is the inverse of the average path lengths from a node to each other node and degree centrality is the number of neighbors for each node [29], [37], [55]. Eigenvector centrality assigns relative scores to all nodes in the network based on the idea that connections to high scoring nodes contribute more to the score of the focal node than equal connections to low scoring nodes [37], [56]. Centrality measures are computed using the *igraph* package [57] implemented in the R version 2.13.1 [58].

### Neutrality and Strength of Species-sorting

We calculate a neutrality index by dividing the variance in species relative abundance among replicated runs for a scenario with species-sorting by the variance among replicates for a neutral model with the same landscape structure [15]. The index ranges from 0 to 1, with a value of 0 for community dynamics dominated by species-sorting and 1 for neutral dynamics (see details in Gravel *et al.* [15]).

We use a conceptually similar approach to measure the strength of the association between species distribution and the environment. We compare simulated results to the expected distribution with perfect species-sorting (i.e. when the species with highest *λ_j,x,t_* wins the competition). We record 1 for a site that is occupied by the best competitor and 0 if by a weaker one. We get an index for the strength of species-sorting at one locality by calculating the number of sites occupied by the best competitor divided by the local community size (*L* ^2^).

### Simulations

We investigate the effects of node centrality in the metacommunity and of environmental heterogeneity with simulations for the following combinations of parameters: (1) species-sorting vs. neutral (2) with or without speciation and (3) three levels of network connectance (*r = *0.15, 0.25 and 0.35, see Fig. S1) (4) four levels of dispersal probability (*m = *0.0001, 0.001, 0.01 and 0.1) (5) seven different levels of environmental heterogeneity (*E_range_* = 0.5, 1.0, 1.5, 2.0, 2.5, 3.0 and 3.5). 20 replicates of 10^6^ time steps were run for each simulation scenario. Speciation rate is set at 0.0001 and kept constant in all simulations. The niche optima of new species are assigned from [*μ_1_, µ_M_*]. Metacommunity size is fixed at *n* = 50 and local community size at *L* = 50, *d* = 0.1, *X* = 50 and *σ* = 1.

## Results

### The Influence of Node Position

**Figure 1:**
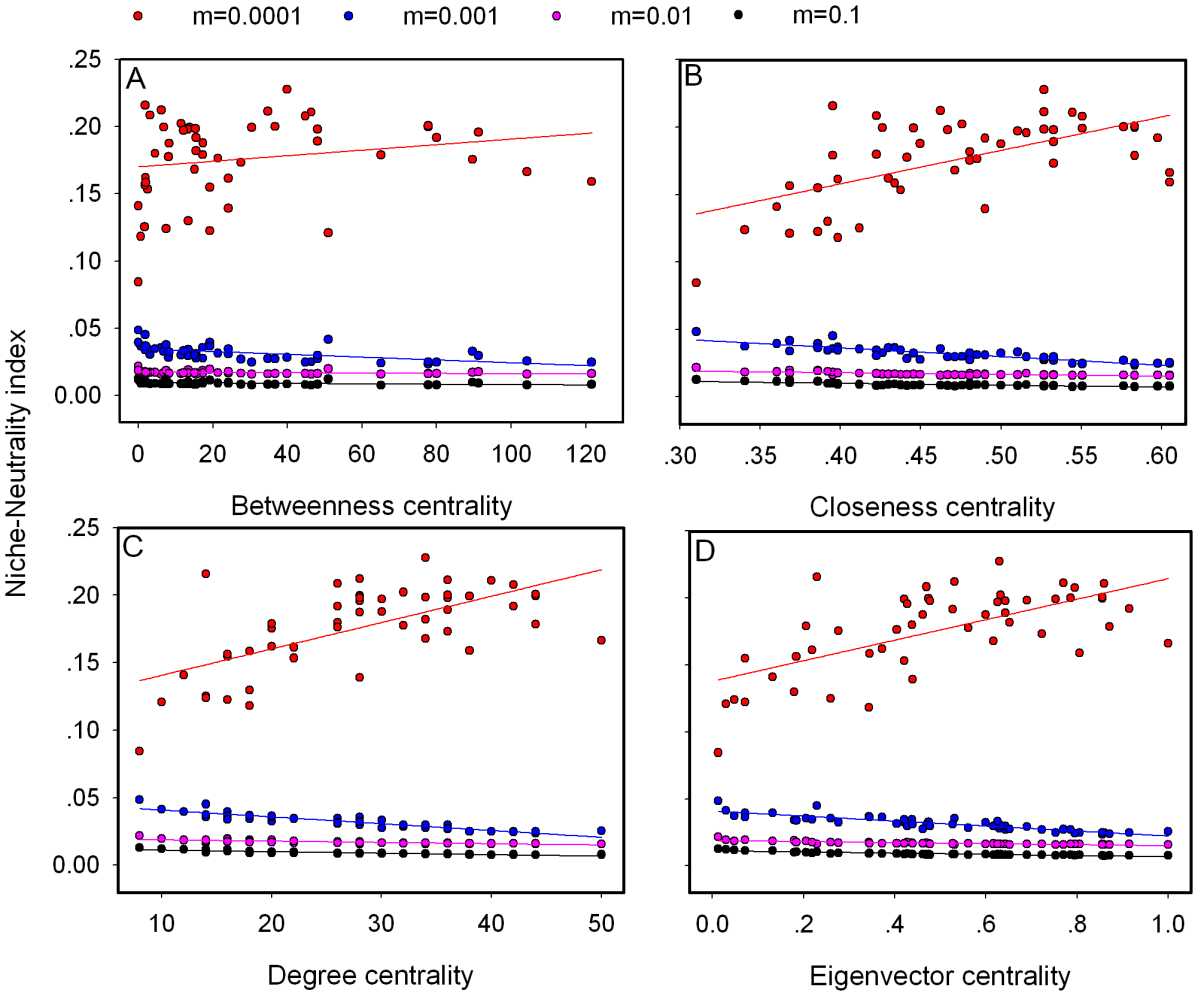
Effect of four centrality metrics in network metacommunity on the niche-neutrality index. There is different migration rates and network connectance *r = *0.35. Each data point is averaged for 20 replicates.

**Figure 2:**
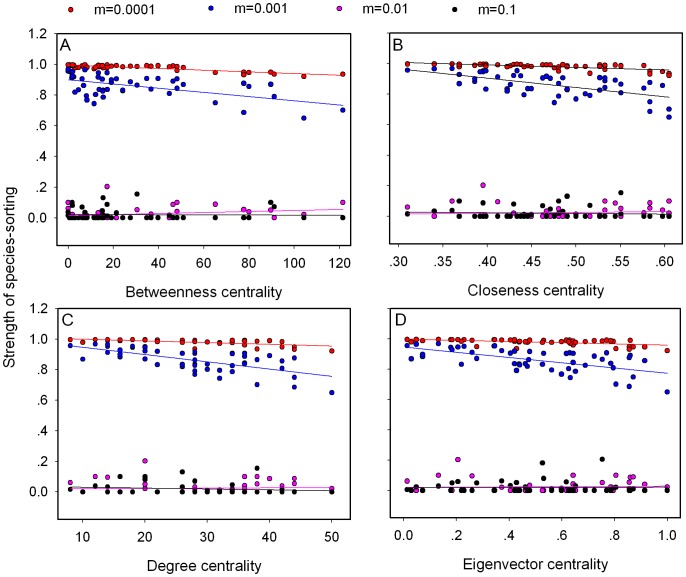
Effect of four centrality metrics on the strength of species sorting. Each data point is averaged for 20 replicates.

We find that local species richness increases with all metrics of node centrality and immigration (Fig. S2). The relationship between node centrality and the neutrality index interacts with immigration (Fig. 1- for simplicity, we only present the results of *r* = 0.35 in the main text, simulations with other values of *r* are attached as Supplementary Information). Neutrality (the variance among replicated runs) increases with node centrality at low immigration, while the opposite is found at high immigration. Species-sorting is strongest for peripheral nodes and lowest for central ones at low immigration, while it is systematically low, independently of node centrality, at high immigration (Fig. 2). The relationship between species-sorting and centrality however quickly saturates at high immigration, as there is no more difference between *m* = 0.01 and *m* = 0.1.

### The Influence of Environmental Heterogeneity

**Figure 3:**
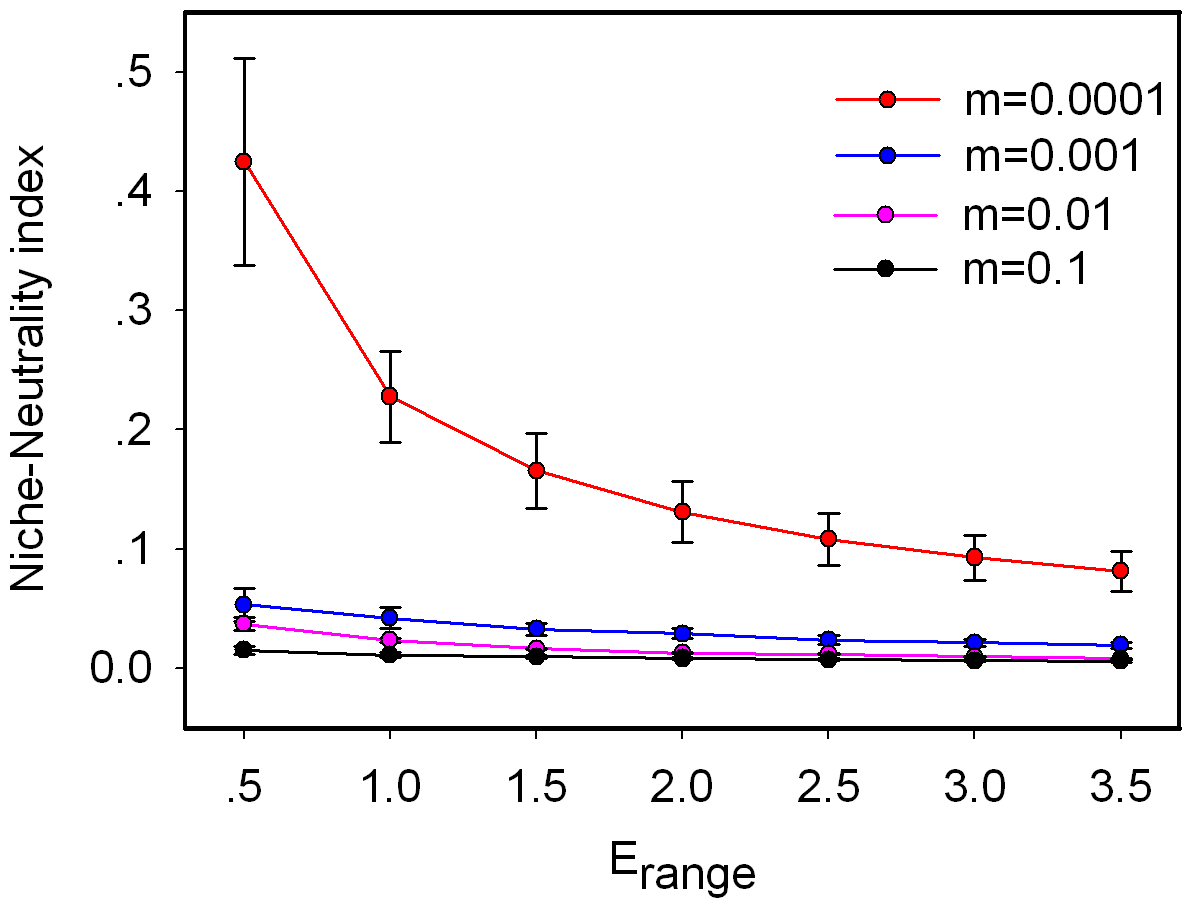
Effect of environmental heterogeneity on the niche-neutrality index. Error bars represent standard deviation over 20 replicate runs.

**Figure 4:**
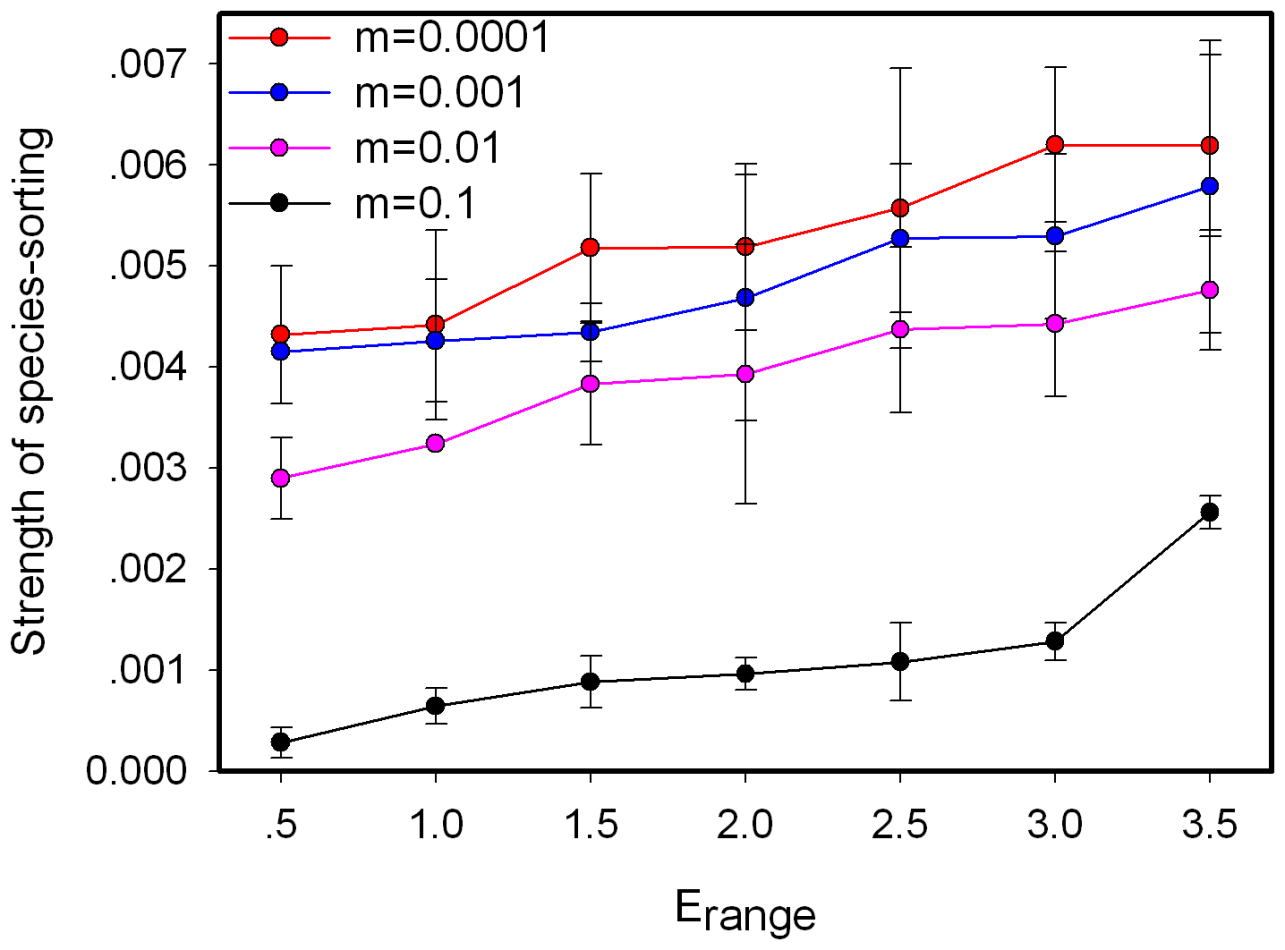
Effect of environmental heterogeneity on the strength of species sorting. Each data point is means for 20 replications.

We find that neutrality decreases with environmental heterogeneity (*E_range_*) at all values of immigration (Fig. 3). There is thus a better match between environmental conditions and species distribution with increasing environmental heterogeneity. Meanwhile, the strength of species-sorting increases with environmental heterogeneity at all values of immigration (Fig. 4).

## Discussion

Our results show that the spatial structure of the metacommunity (i.e. the way localities are connected by dispersal and the environment heterogeneity) has considerable impacts on the strength of species-sorting, ecological drift, and ultimately, species distribution. Spatial contingencies such as the position of a local community in the landscape and the distribution environmental conditions influence the ability of a species to track its favorable environment. Species-sorting is typically considered a process [59] whereby here it is quantified as an emergent pattern – the realized correspondence between environment and species distributions. We emphasize two conclusions from our analysis. First, neutrality increases and the strength of species-sorting decreases with the centrality of a local community when the average dispersal is relatively low, whereas the opposite relationship is found at higher dispersal. Second, neutrality decreases and the strength of species-sorting increases with the variance of environmental conditions.

Both the average dispersal rate and the position of local communities contribute to the connectivity matrix [60]. We consequently find that species richness increases with both the immigration probability and node centrality (Fig. S2; [37]), as observed in previous studies on the mass effect [61]. High immigration and centrality promote the mass effect because they increase the influx of inferior competitors and therefore the likelihood of their recruitment. The influence of the position of a local community in the landscape on the strength of species-sorting and ecological drift however differs with the average immigration (Fig. 1, Fig. 2, Fig. S3 and Fig. S4).

Under low immigration and in peripheral nodes, there is no mass effect and therefore the best competitor systematically (deterministically) wins the competition. Species-sorting is therefore strong and neutrality low. At central nodes there is a significant mass effect occurring, preventing the top competitor to win. Species identity could however be deterministic as well (and therefore both neutrality and species-sorting low), for instance in a sink node constantly receiving the same influx of migrants from the source node. If there are enough spatial refuges (peripheral nodes where a species could deterministically outcompete other ones), then the neutrality at the regional level will remain low because they will prevent drift to extinction at the regional scale. The situation changes at high immigration because species are moving rapidly throughout the landscape and often the best competitors are outcompeted by chance alone. Regional diversity reduces [20] and the amount of drift increases because there is a much higher niche overlap [15]. Peripheral nodes are subject to a lot of stochasticity because the drift will be maximal (no immigration to prevent random extinction). On the other hand, central nodes will be dominated by the most abundant species at the regional level and the composition might thus appear more stably. The species-sorting is consequently much lower at high immigration.

Our results show that despite species-sorting and ecological drift being conceptually opposite, this relationship does not always hold. The match between species distribution and the environment could obviously only be low under neutral dynamics. We have however found that in the presence of a strong mass effect, there will be low species-sorting by definition (top competitors are replaced by inferior ones). Neutrality might however remain low provided there are locations where some species deterministically win competition and therefore act as constant sources of immigrants. The emergent mixture of coexistence mechanisms found across the landscape is perhaps the most important contribution from our study. These results could have only been found with spatial heterogeneity in the environment and in the connectivity matrix, promoting a variable distribution of sources (refuges) and sinks.

We also find that increasing the environmental heterogeneity strengthens species-sorting (Fig. 3, Fig. 4, Fig. S5 and Fig. S6). The average niche overlap decreases with larger difference between adjacent patches, thereby making it easier for each species to find and fight for their favorable habitat. Snyder and Chesson [51] investigated the joint impacts of dispersal and environmental heterogeneity on coexistence and found that short-range dispersal provide a significant advantage when environmental structure is permanent. The range of environmental variation determines how many niches there will be for a given niche breadth and the effect of dispersal to coexistence depends strongly on environmental variation [51]. Diversity is consequently impacted by environmental heterogeneity (Fig. S7), as studied by Schwilk and Ackerly [43].

Our study adds to current metacommunity theory by considering that beyond the effect of average dispersal among local communities, its variance might also influence biodiversity. We do not vary immigration probability over space in our simulations, thus spatial variance in dispersal nonetheless arises in our simulations from two independent drivers. First, the spatial network structure (the connectivity matrix) determines the relative importance of dispersal among local communities and the structure of the metacommunity [37]–[39]. Second, spatial heterogeneity of the environment determines species distribution and thereby the composition of the flow of migrants at a given location [62]. With increasing variance in dispersal, some local communities could be neutral or subject to a mass effect, while others might experience a strong species-sorting. There is consequently spatial variance in the coexistence mechanisms at play. For instance, diversity in local communities with low centrality is maintained by species sorting, while diversity in central communities is maintained by neutral drift and the mass effect. This spatial variance of coexistence mechanisms should promote the regional diversity by reducing the regional similarity constraint [20]. If there is variance in dispersal, metacommunity could experience much higher average dispersal without having the homogenizing effect of dispersal [20], [63], [64].

Species distribution models are important tools to study the distribution of species and forecast their response to environmental changes. Recently, Boulangeat et al. [65] proposed a new framework integrating abiotic constraints, dispersal limitations and biotic interactions to predict the species distribution and abundance. The increased performance of their integrated model is indeed attributable to explicit accounting of dispersal and environmental heterogeneity. In our model, we consider both the connectivity matrix and the environmental heterogeneity, and they interact to affect the species distribution model. Without environmental heterogeneity, our model converge to the neutral model with network structured metacommunity [37]; while species will be sorted out according to the environmental condition in absence of connectivity matrix [66].

## Supporting Information

Figure S1 **The structure of metacommunity under different connectance** ***r*** **= 0.15 (top),** ***r*** **= 0.25 (middle) and** ***r*** **= 0.35 (bottom).**
(TIF)

Figure S2 **Effect of four centrality metric in network metacommunity on the number of species.**
(TIF)

Figure S3 **Effect of four centrality metrics on the niche neutrality index with** ***r*** **= 0.15 and** ***r*** **= 0.25.**
(TIF)

Figure S4 **Effect of four centrality metrics on the strength of species sorting.** The network connectance *r* = 0.15 and *r* = 0.25, and migration rate is different.
(TIF)

Figure S5 **Effect of environmental heterogeneity on the niche-neutrality index with** ***r*** **= 0.15 and** ***r*** **= 0.25.**
(TIF)

Figure S6 **Effect of environmental heterogeneity on the strength of species sorting with** ***r*** **= 0.15 and** ***r*** **= 0.25.**
(TIF)

Figure S7 **Relationship between α-, β- and γ-diversity and environmental heterogeneity.** By setting *E_range_* to 0, species niche optima become identical and the model converges to a neutral model. Each data point is the mean of 20 replications for each local community. Error bars represent the standard deviation.
(TIF)
