# Supplementary figures and images for: Spatial Structures of the Environment and of Dispersal Impact Species Distribution in Competitive Metacommunities
Source: PLoS One. 2013 Jul 18;8(7):e68927. doi: 10.1371/journal.pone.0068927 (PMC3715503; doi:10.1371/journal.pone.0068927)

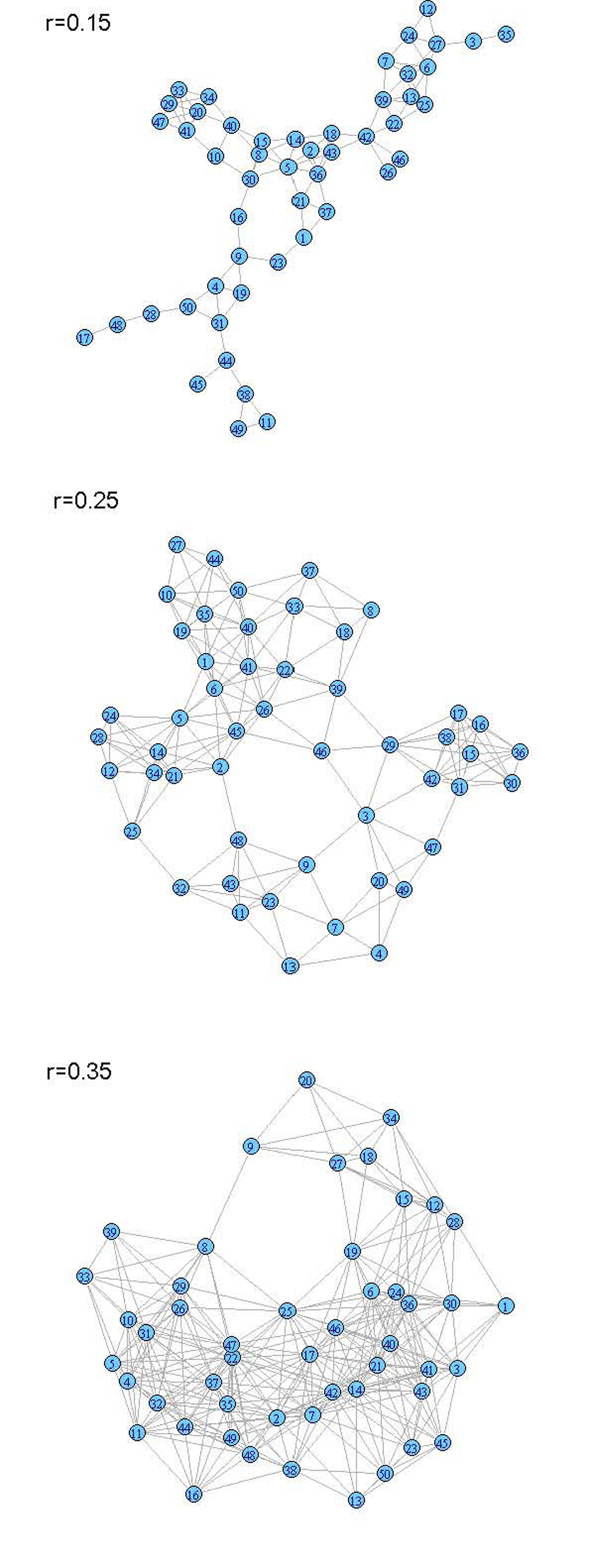

Supplement: Figure S1 — The structure of metacommunity under different connectance r = 0.15 (top), r = 0.25 (middle) and r = 0.35 (bottom). (TIF) [file pone.0068927.s001.tif]

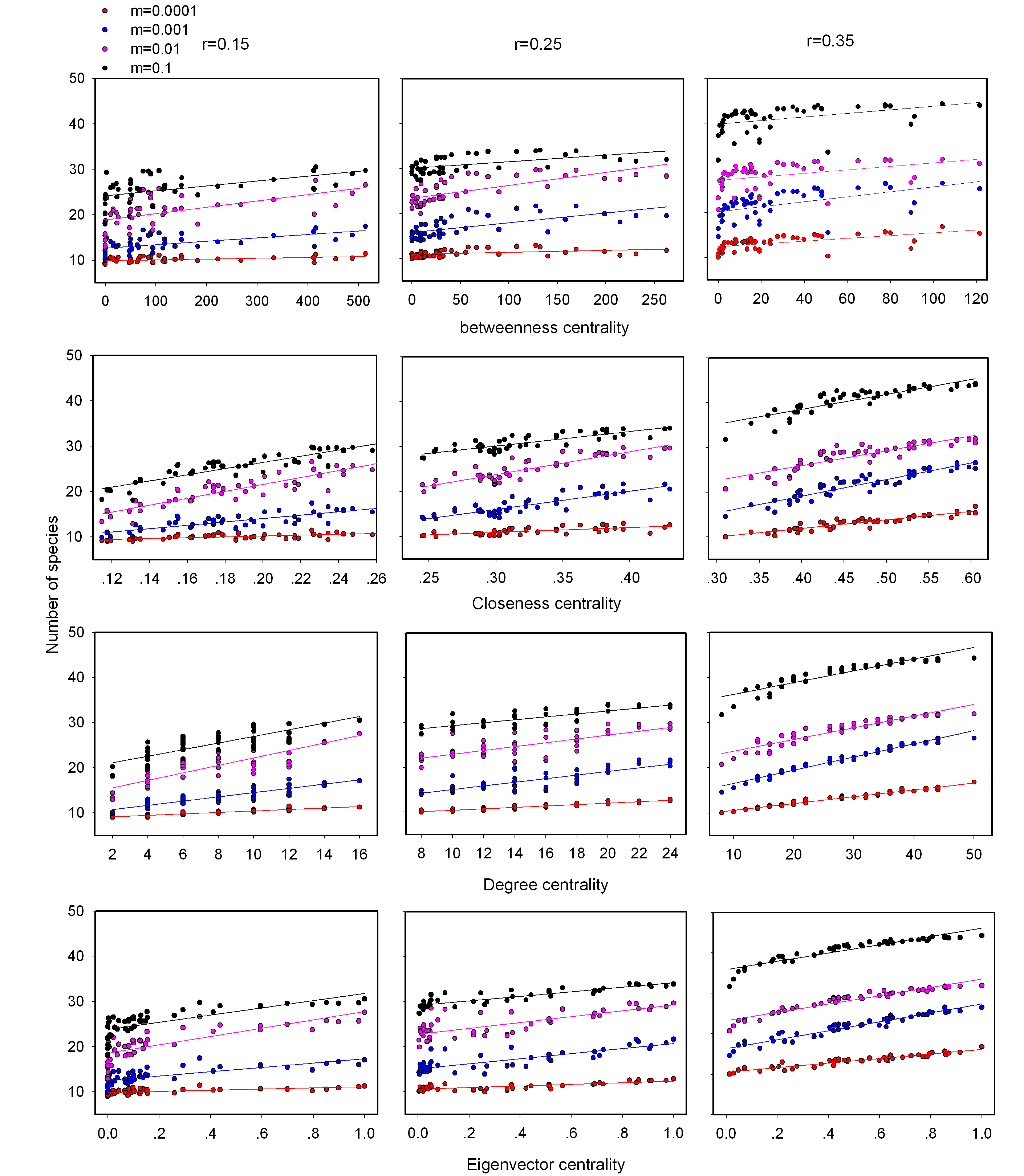

Supplement: Figure S2 — Effect of four centrality metric in network metacommunity on the number of species. (TIF) [file pone.0068927.s002.tif]

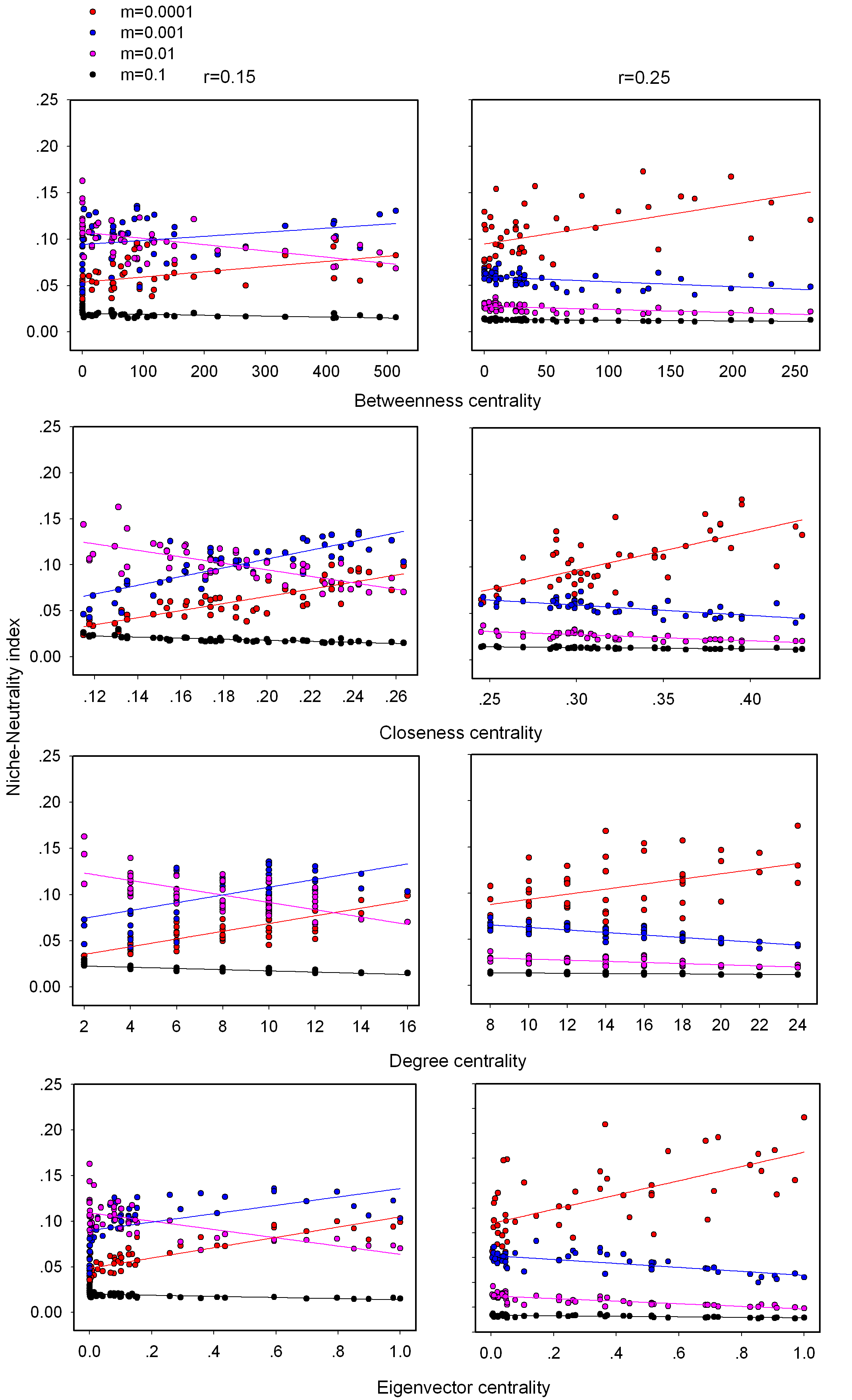

Supplement: Figure S3 — Effect of four centrality metrics on the niche neutrality index with r = 0.15 and r = 0.25. (TIF) [file pone.0068927.s003.tif]

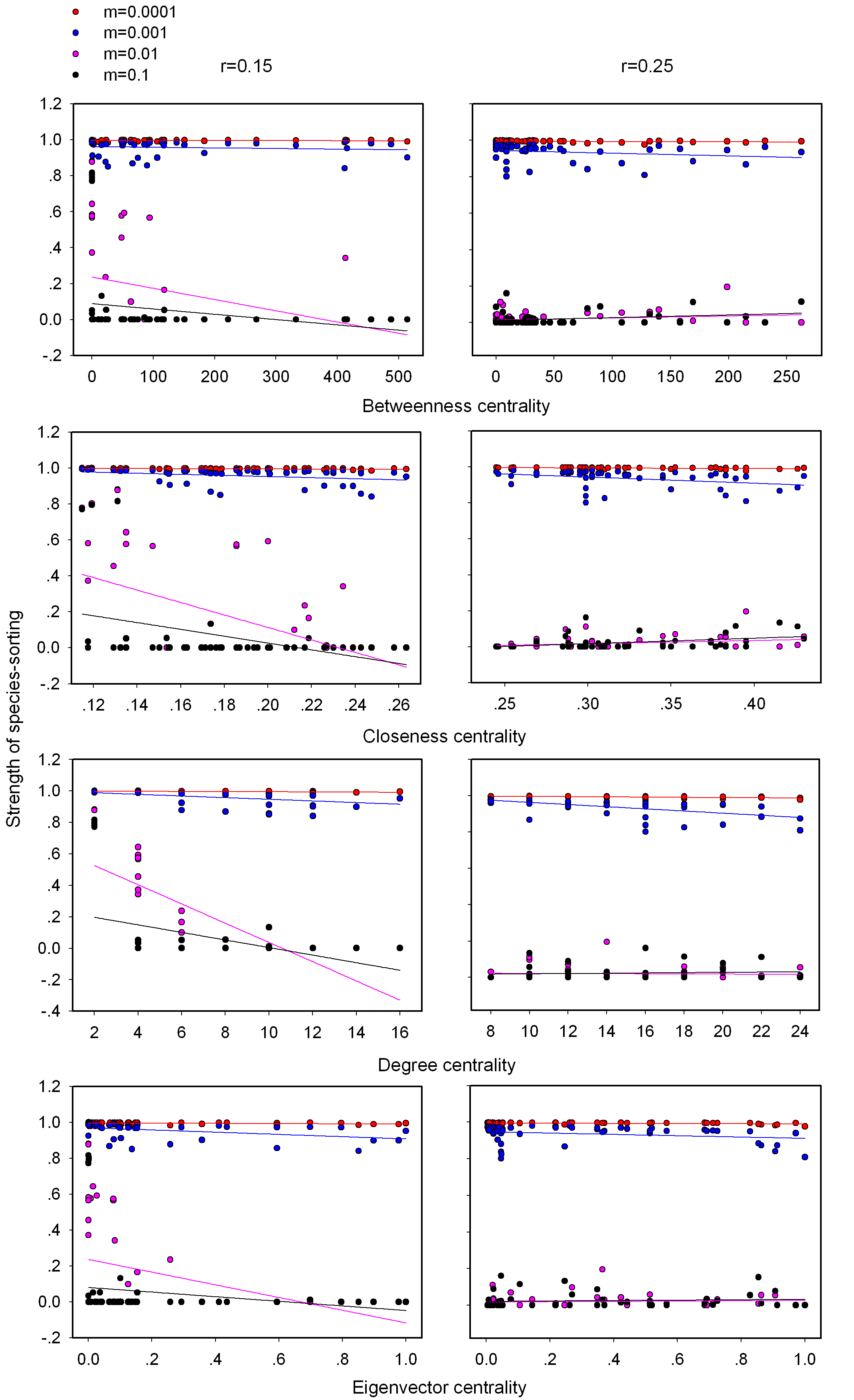

Supplement: Figure S4 — Effect of four centrality metrics on the strength of species sorting. The network connectance r = 0.15 and r = 0.25, and migration rate is different. (TIF) [file pone.0068927.s004.tif]

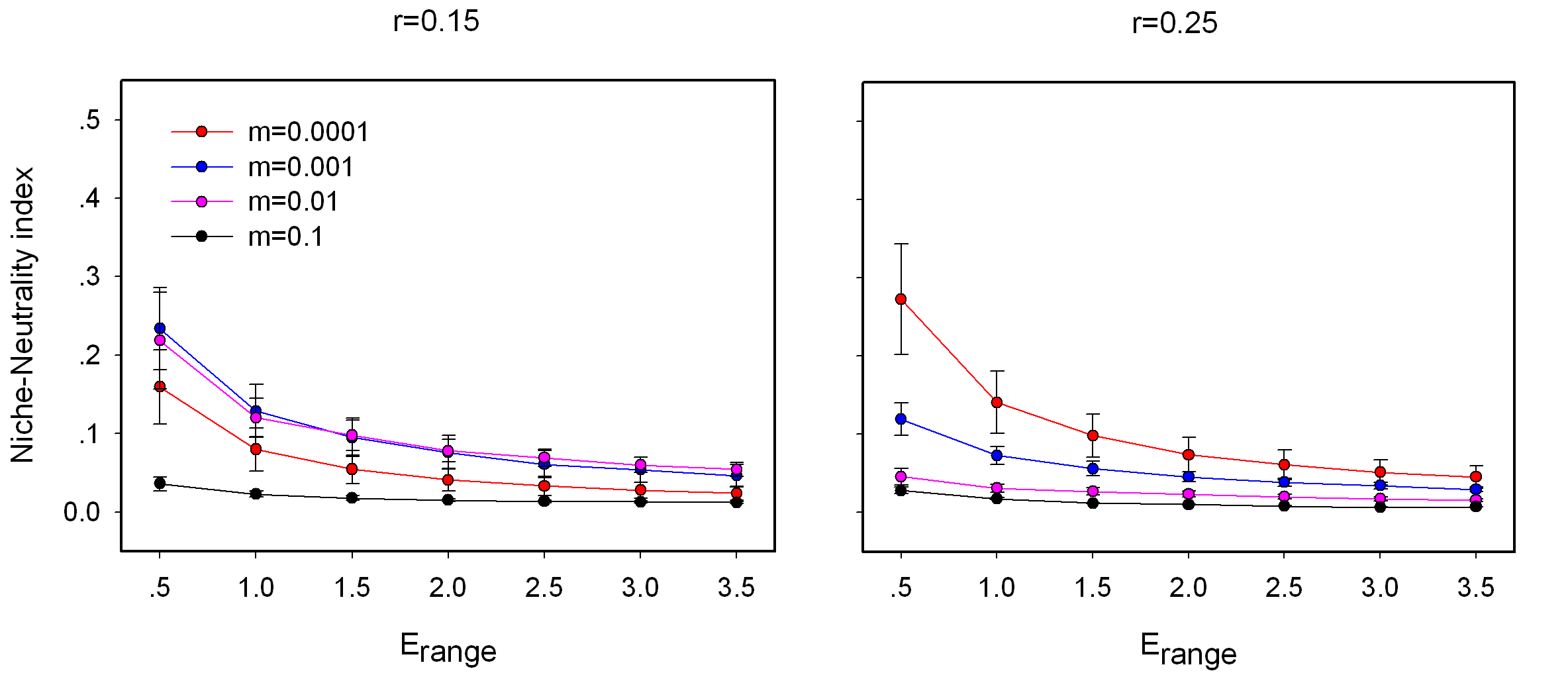

Supplement: Figure S5 — Effect of environmental heterogeneity on the niche-neutrality index with r = 0.15 and r = 0.25. (TIF) [file pone.0068927.s005.tif]

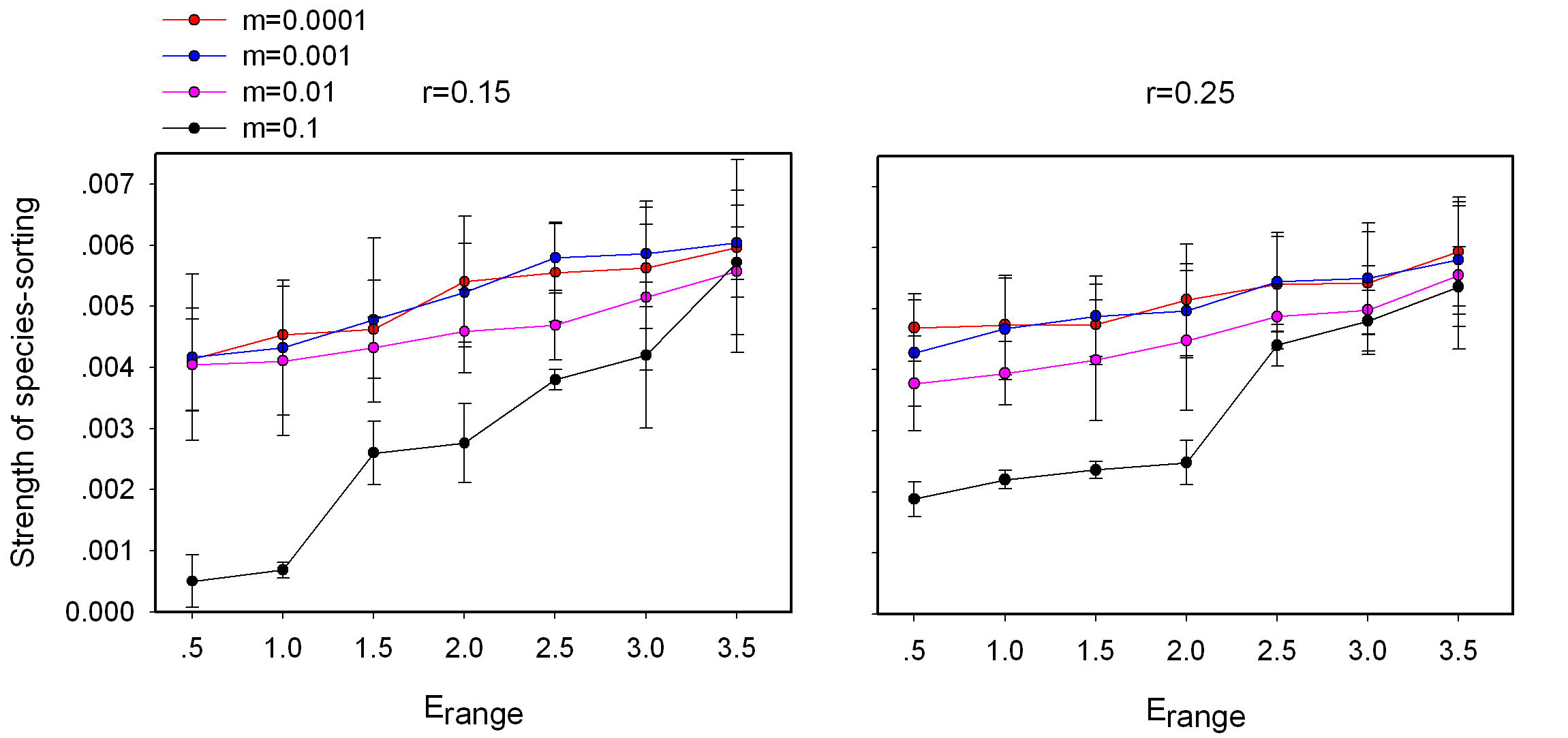

Supplement: Figure S6 — Effect of environmental heterogeneity on the strength of species sorting with r = 0.15 and r = 0.25. (TIF) [file pone.0068927.s006.tif]

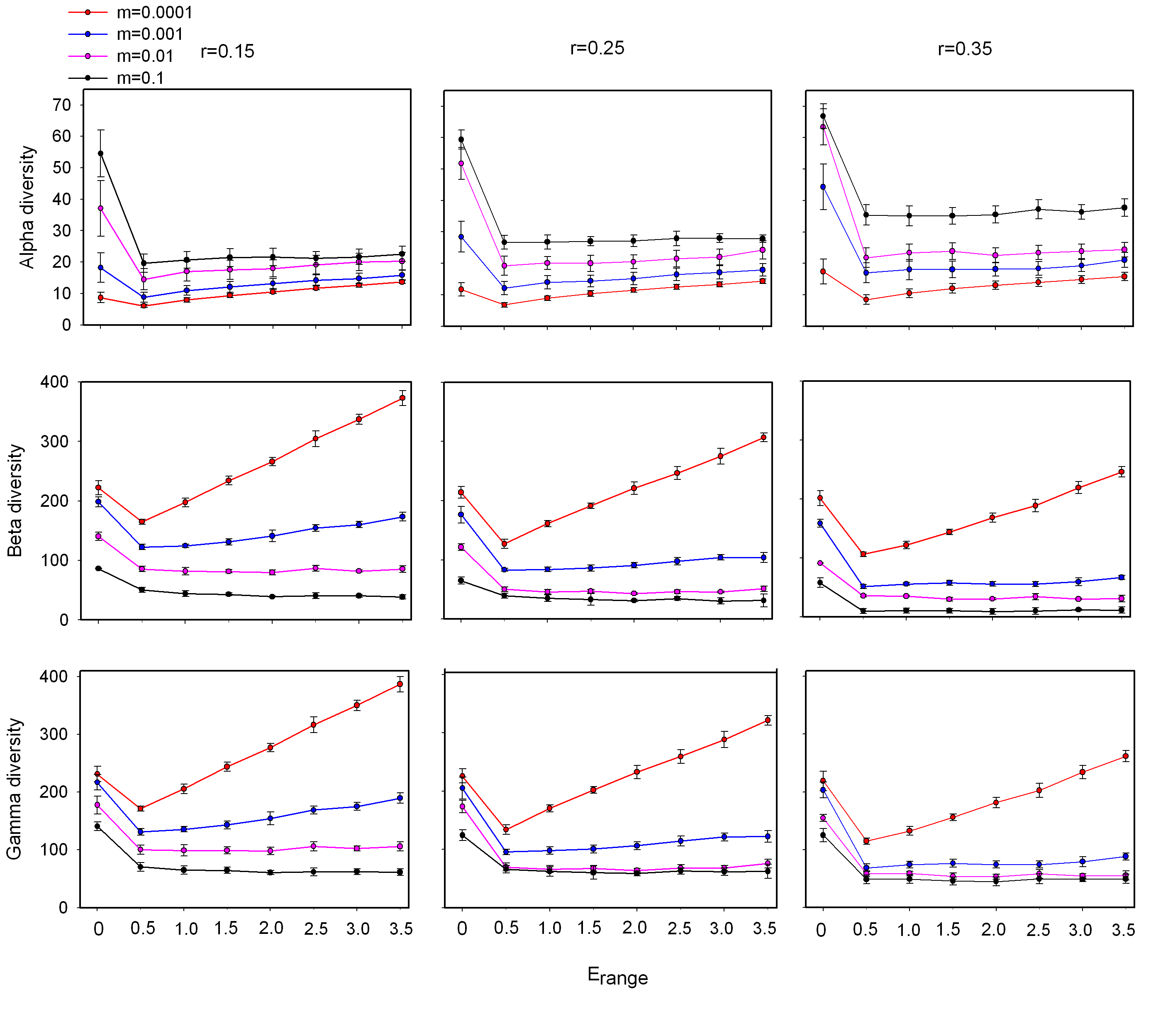

Supplement: Figure S7 — Relationship between α-, β- and γ-diversity and environmental heterogeneity. By setting Erange to 0, species niche optima become identical and the model converges to a neutral model. Each data point is the mean of 20 replications for each local community. Error bars represent the standard deviation. (TIF) [file pone.0068927.s007.tif]
